# Penetrating Wounds of the Chest

**Published:** 1896-05

**Authors:** 


### PENETRATING WOUNDS OF THE CHEST.

Huget and Peraire (*Revue de Chirurgie*,) after a thorough discussion of this question, and a number of interesting cases, come to the following conclusions:

1. That it is absolutely necessary in these cases to treat the patients at the time and in the place where the accident occured without waiting to carry them to a hospital. The jolting and movements inevitable during transportation are very liable to produce a hæmoptysis, or increase one already present and so cause death.

2. The tendency to syncope should be respected, as it favors hæmostasis. Consequently, subcutaneous injections of ether should rarely be given unless the depression is marked. The injection to be preferred in these cases is of caffeine or of artificial serum.

3. The treatment of these wounds by ordinary means is useless in the majority of cases unless the patient is put absolutely at rest. The most perfect repose is secured by immobilizing the thorax by means of adhesive plaster and a well-applied bandage.

4. This method of treatment does not, of course, preclude the use of antiseptic treatment of the wound, with ligation of arteries, suturing of the wound, and the use of compressive and occlusive antiseptic dressings.

5. In case a hæmothorax arises from the injury of a large vessel, the treatment should be one of "aimed expectation," and is entirely dependent upon the conditions present. Symptoms and complications are the guides of treatment. Thoracentesis should not be practised unless the extravasation of blood is very large and death from dyspnœa is threatened.

6. In these cases the toilet of the person, after the wound has been dressed, should be avoided.—*International Medical Magazine.*

